# Can Preference for Oviposition Sites Initiate Reproductive Isolation in *Callosobruchus maculatus*?

**DOI:** 10.1371/journal.pone.0014628

**Published:** 2011-01-31

**Authors:** Emma Rova, Mats Björklund

**Affiliations:** Department of Animal Ecology, Evolutionary Biology Centre, Uppsala University, Uppsala, Sweden; Aarhus University, Denmark

## Abstract

Theory has identified a variety of evolutionary processes that may lead to speciation. Our study includes selection experiments using different host plants and test key predictions concerning models of speciation based on host plant choice, such as the evolution of host use (preference and performance) and assortative mating. This study shows that after only ten generations of selection on different resources/hosts in allopatry, strains of the seed beetle *Callosobruchus maculatus* develop new resource preferences and show resource-dependent assortative mating when given the possibility to choose mates and resources during secondary contact. The resulting reduced gene flow between the different strains remained for two generations after contact before being overrun by disassortative mating. We show that reduced gene flow can evolve in a population due to a link between host preference and assortative mating, although this result was not found in all lines. However, consistent with models of speciation, assortative mating alone is not sufficient to maintain reproductive isolation when individuals disperse freely between hosts. We conclude that the evolution of reproductive isolation in this system cannot proceed without selection against hybrids. Other possible factors facilitating the evolution of isolation would be longer periods of allopatry, the build up of local adaptation or reduced migration upon secondary contact.

## Introduction

During the last decade, interest in speciation has been renewed as a major topic of research [1], [2]. Many models of speciation have been analyzed based on various premises and ecological situations, including sympatric speciation [1], [3], secondary contact and reinforcement [4], [5]. One set of models combines the process of divergent selection for resource use with the evolution of assortative mating. If there is selection for differential resource use, then this may foster selection for preferred matings between individuals using the same type of resource, as random mating would result in unfit hybrids [6]-[8]. This requires linkage disequilibrium to build up between loci determining resource use and loci determining mating preference. Alternatively, the mechanism could involve a so called ‘magic trait’ [1], i.e. a set of loci that pleiotropically determine both mating and resource preference [9], [10]. Most of these models assume that a linkage between preference for a specific host and performance on that same host must exist, i.e. that there needs to be a fitness-cost to choosing the wrong host for such a preference to evolve in the first place. However, it has been shown that a host preference can indeed evolve in the absence of local adaptation and that assortative mating, and possibly reproductive isolation can result as a consequence [11].

One possible scenario where the above mentioned models might apply is when a host shift occurs in phytophagous insects [12]-[14]. Here a preference for a new resource might evolve and with that, a preference for mates. This might readily happen under allopatric conditions, or under sympatric or parapatric conditions, when mating occurs in the host plant. But what would happen if two strains selected on separate host plants in allopatry come into secondary contact (in sympatry or parapatry)? For speciation to occur in such a case, linkage between mate and resource preferences must be maintained in sympatry to uphold the genetic integrity of the diverging populations [15]. The outcome depends on a combination of the strength of selection for preference for each host plant, and the strength of assortative mating. Selection acting on both these traits facilitates divergence to a larger extent than selection for either trait independently. However, selection for one of these traits may be sufficient to generate reproductive isolation between populations inhabiting different hosts [3].

In this paper, we experimentally test some key predictions derived from the models above, such as the development of host use (preference and performance) and assortative mating, and in particular the interaction between the two. By using allopatric lines of seed beetles raised on different food resources for ten generations, we can test if *i*) linkage between host preference and mating preference can arise and *ii*) whether this linkage can be maintained under sympatric conditions without any apparent selection against random mating. We test this by comparing mating frequencies of beetles, raised on different resources/hosts, over successive generations in secondary contact.

## Materials and Methods

### Laboratory population

In this study we used a laboratory population of the Cowpea weevil, *Callosobruchus maculatus (Fabr.)*, originating from Brazil and kept on black-eyed beans, *Vigna unguiculata (L.)*, in various labs for about 20 years. The species is a cosmopolitan pest of stored legumes (*Fabaceae*), particularly beans of the genus *Vigna* [16]. It has non overlapping generations, and a generation time of approximately 28 days. Prior to the experiment, strains were kept at constant large population sizes (several hundreds of individuals) on black-eyed beans. During the experiment, populations were maintained in dark climate chambers at 26°C and 55% relative humidity.

The species has two naturally occurring colour morphs, one brown and one black. When performing reciprocal crosses between the black and brown colour morphs in *C. maculatus*, Eady [17] found that all resulting offspring were of the intermediate type and that F1 crosses yielded a distribution of morphs in concordance with expectations from colour polymorphism being controlled by a single locus with two alleles, even though the exact details of the genetic background are unknown. However, for the purposes of this experiment we assumed a a simple genetic background.

### Selection

We employed artificial selection for the two different colour morphs (black and brown), and allowed natural selection on two alternative food resources (black-eyed (BE) beans and mung (M) beans, *Vigna radiata (L.)*). The beans differ in size, water content, hardness and texture of the seed coat, with BE-beans being larger, containing more water and having softer seed coats with rough surfaces compared to M-beans. In the subsequent experiments, we used colour as a marker for convenience in conducting crosses between the BE and M adapted beetles. Since all matings took place in dark climate chambers, it seems unlikely that colour would interfere with mating preferences in this study. We selected the black morph for black eyed beans and the brown morph for mung beans. In total, the experiment comprised 18 lines (nine replicates for each resource). Selection lasted for ten generations during which we selected the ten most distinct (brown or black) males and females from each generation from a population of several hundred individuals per line. Thus, during selection, each new generation started with a population size of 20 individuals.

### Test of resource preference

At the end of the selection process, we separately introduced each morph type (black and brown) to a mixture of resources, to test for resource preference. The mixture contained the resource that the particular morph type had been reared on during the selection process, and a newly introduced resource. We placed ten newly hatched virgin females together with ten newly hatched virgin males on 40 g of beans (20 g BE and 20 g M), whereupon mating females were allowed to choose on which type of resource to deposit their eggs. After egg-laying, we separated the two resources and counted the eggs laid on each resource. We then compared the proportion of eggs laid on M beans by individuals reared on M beans (MM), to the proportion of eggs laid on M beans by individuals reared on BE beans (BEM), and vice versa. We did this for all lines on each of the two resources.

### Environmental and maternal effects

To remove maternal effects, after the selection period we introduced ten females and ten males reared on BE beans to the alternative resource, M beans, for one generation. We then introduced ten females and ten males from the following generation emerging from the M beans to a mixture of the two alternative resources. All mated females were, as in the preceding experiment, allowed to choose where to lay their eggs. After egg-laying, we separated the resources and counted the number of eggs. We then compared the number of eggs on the two different host plants. If maternal effects were in fact an issue, the number of eggs laid would be significantly higher on the alternative resource (M) compared to the original one (BE), as the progeny would choose the same host plant as their mother.

### Test for assortative mating

To test whether assortative mating evolved, we placed colour morphs selected on alternative resources together on a mixture of the two bean types. Populations were mixed in a pair wise fashion, such that line1 on M beans were mixed with line 1 on BE beans and so on. 20 virgin females and males from each morph type were selected randomly, resulting in 80 individuals on 80 grams of beans (40 g of M beans and 40 g of BE beans). This was done for eight generations, with the exception that in all generations following the first, 20 individuals from each sex were selected at random from the two resources to mirror the existing morph frequency of each resource. The two different morph types were allowed to mate freely and females were allowed to choose on which type of resource to deposit their egg load. After egg-laying, we separated the resources and upon emergence of the F1 generation, we counted the distributions of the different morphs on each resource.

To test for assortative mating, we looked for deviations from Hardy-Weinberg proportions in colour morph frequencies. In a large population with no selection, random mating, no mutations and no gene flow, genotype frequencies can be calculated from gene frequencies [18]. In our experimental settings the only possible reasons for deviations from Hardy-Weinberg is sampling error (limited population), selection and non-random mating. We tested explicitly for the presence of disruptive selection (see below). In the absence of selection, significant deviations from HW in our experiment can result from either non-random mating or sampling errors. Note that this test does not provide any information on the causes of assortative mating; it simply reveals its presence. As a measure of assortative mating, we used the heterozygote deficiency index, *I*  = 1- Het_obs_/Het_exp_ [1], which is zero in the case of no assortative mating, and one if there is total reproductive isolation. To account for sampling error, we applied a Bayesian approach using WinBugs 1.4.1. [19]. We estimated the multinomial proportions (the two homozygotes and the heterozygote) with a Dirichlet distribution as the prior and used 10^6^ updates, after a burn-in of 10 000 steps. In this way we obtained an estimate of *I* and 95% credibility intervals that accounted for the limited sampling. For a detailed description on the methods, see [20].

### The nature of assortative mating

To discriminate between assortative mating based on the resource used during the selection period and that based on the colour morph, we measured the mate choice of 20 virgin females from the base population toward males from different colour morphs stemming from the selected lines. In one set of trials, a black female was placed together with a brown and a black male in a Petri-dish. In the other set of trials, a brown female was placed together with a black and a brown male in a Petri-dish. Females were allowed to choose which male to mate with. The colour of the male accepted by the female was noted, as was the time elapsed before each mating started and the duration of each mating event.

### Neutrality of colour morphs

To discern whether colour was neutral relative to preference and performance, i.e. that the marker trait itself would not confound the data, the 20 females from the mating experiment above (stemming from the base population unexposed to selection), were each put in a Petri-dish with a mixture of 20 g of M beans and 20 g of BE beans. Females were then allowed to deposit their entire egg loads on the two different substrates. The two different bean types were then separated and eggs and emerging individuals were counted for each female and each bean type. The number of eggs laid per resource served as a measure of preference for that resource and the number of individuals hatched on each resource was used as a measure of performance. Host preference and host performance was then compared between females of different colours.

### Selection against hybrids

In the absence of selection against heterozygotes, a deficit of heterozygotes in comparison to HW expectations would be due to assortative mating alone. Thus, in this last experiment, we tested for possible fitness differences between the three morph types (black homozygote, intermediate heterozygote (first generation hybrids) and brown homozygote) on the two alternative resources. From the lines reared on M beans, we selected ten females and ten males from each morph type and placed them in three different jars respectively on 40 g of M beans. We carried out the same procedure for the lines reared on BE beans, only they were placed on 40 g of BE beans. In each treatment males and females of the same colour morph were allowed to mate with each other and females were allowed to deposit their eggs. We counted and compared the number of eggs laid and the number of adults emerged between the three different morphs on both bean types. Each treatment consisted of two replicates per bean type.

An alternative way of testing for possible selection against heterozygotes is to compare the allele frequencies before and after the test period. Allele frequencies are expected to change to some extent due to drift, but if this change is larger than expected, then selection is a possible explanation. We compared the allele frequencies between successive generations starting with the first generation after secondary contact. The variance in allele frequencies due to drift is given by equation
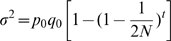
(1)
[19], where *p*_0_ is the initial frequency of allele *p*, *t* is the number of generations and *N* is the population size. This expression is valid for the first generation. For the second generation p1/q1 was used as the new allele frequencies and for generation three, p2/q2, and so on. Population size should be effective population size (*N_e_*), but as we had no information on this, we used a Bayesian approach that we implemented in WinBugs [19]. Here we used a uniform prior for the effective population size ranging between 0.1-1× N. Since between-generation variance in frequencies is a result of both drift and limited sampling, we combined these two sources of variance, with sampling variance estimated in the same way as when we tested the index of assortative mating. We used this combined variance and estimated the expected changes in allele frequencies as being normally distributed with a mean of zero and a variance given by the combined variance. In this way, we estimated the mean and 95% confidence interval for the expected allele frequencies between generations and compared that to the observed change in frequencies.

## Results

**Figure 1:**
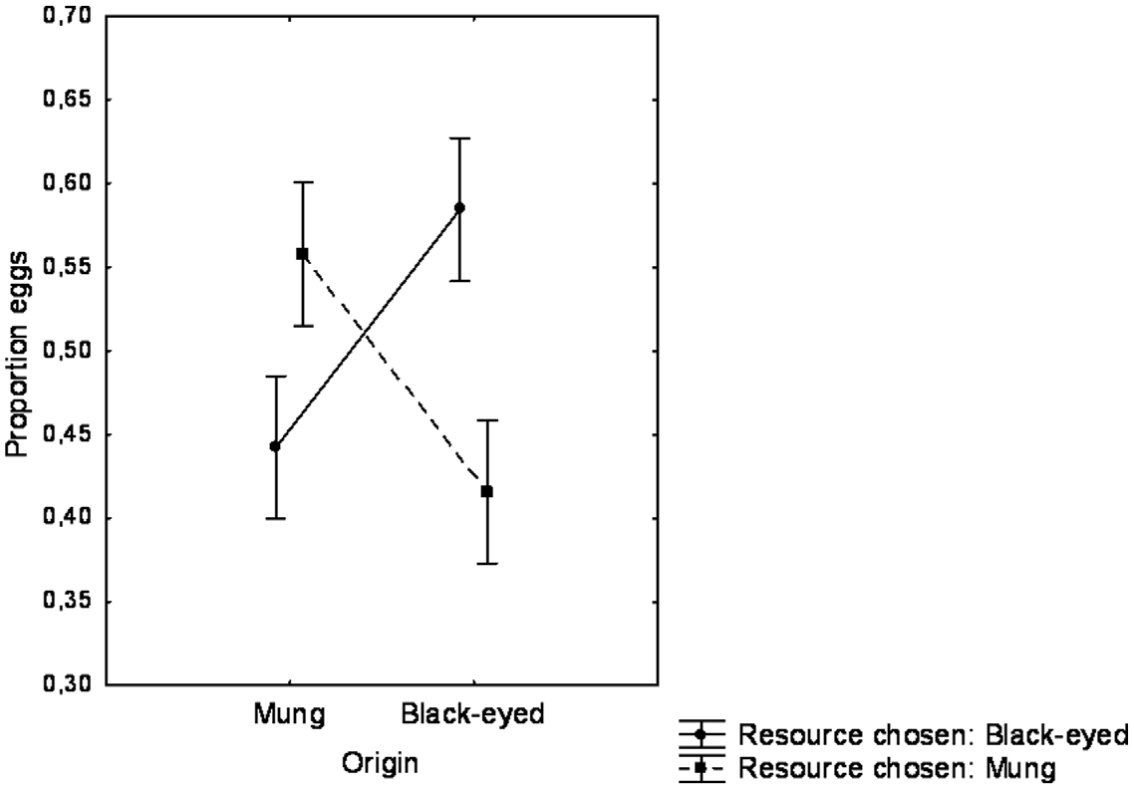
The proportion of eggs laid. The proportion of eggs (±95% confidence interval) laid by females in the different treatments. Origin refers to the bean type females were raised on for ten generations, and resource chosen refers to the bean type they chose to lay their eggs on when offered a mixture of the two bean types as oviposition substrate.

After ten generations of selection on a new resource, we found a strong preference for novel egg laying sites, and a significant reduction in preference for the ancestral host in lines where females were allowed to choose where to deposit their egg loads. Overall, the interaction between origin and resource chosen was highly significant (factorial ANOVA, F_1, 32_ = 16.6, P = 0.00029: Figure 1). Lines selected on M beans laid a significantly larger proportion of eggs on M beans than did lines selected on BE beans (Fishers LSD test, P = 0.00014, df = 32; Figure 1). Lines selected on BE beans laid a significantly larger proportion of eggs on BE beans than did lines selected on M beans (Fishers LSD test, P = 0.0043, df = 32; Figure 1).

After ten generations of selection on the two different host beans BE and M, we introduced individuals from the BE lines to the alternative resource, M beans, for one generation. We found no preference for the novel resource after one generation. In fact, the number of eggs laid on the resource selected for prior to this experiment (BE) was significantly higher compared to the number of eggs laid on the newly introduced one (M) (BE 344.4±18.75, M 236.3±25.68, mean ± SE number of eggs, *P* = 0.0056, Mann-Whitney U-test).

**Figure 2:**
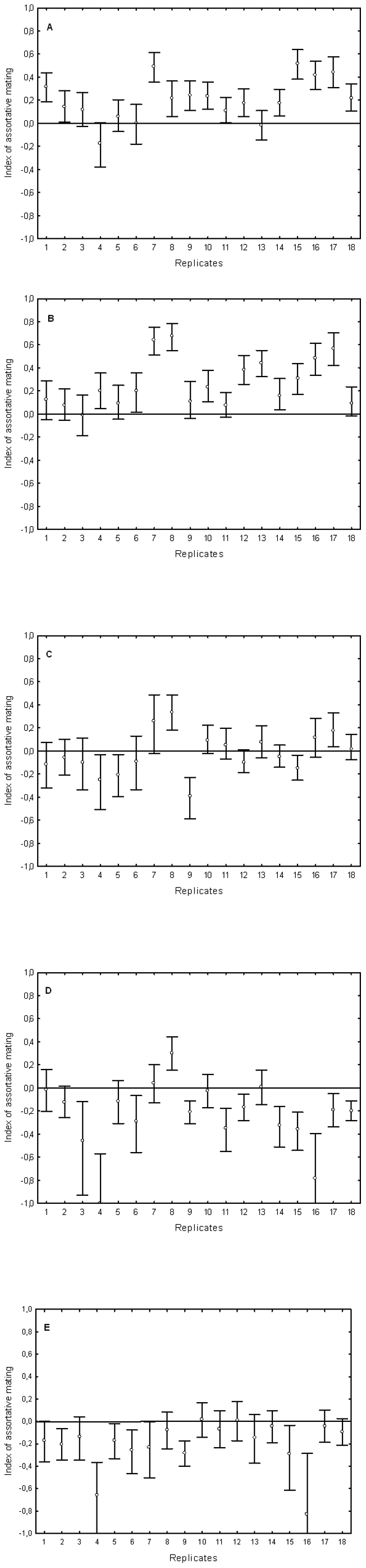
Mean index of assortative mating. Mean (±95% confidence interval) index of assortative mating for all 18 replicates. Replicates 1–9 were run on BE beans, and replicates 10–18 on mung beans. a) Generation 1, b) generations 2, c) generation 3, d) generation 4, e) generation 8.

In the first generation after secondary contact, when the two morphs were kept together on a mixture of the two resource types, we found a significant deficit of heterozygotes in 13 out of 18 cases (mean *I  = *0.20±0.043, mean ± SE heterozygote deficiency index; for details on each line, see Figure 2a where intervals not including zero indicate that the *I*-value is significant). In the second generation, eleven lines showed a significant deficit of heterozygotes (mean *I*  = 0.27±0.050; Figure 2b). In the third generation, four lines showed a significant deficit of heterozygotes, while two lines showed a significant excess of heterozygotes (mean *I  = *−0.02±0.043; Figure 2c). In the fourth generation one line showed a significant deficit of heterozygotes, and eleven lines showed a significant excess of heterozygotes (mean *I  = *−0.24±0.071; Figure 2d). We terminated the experiment after eight generations, and seven lines showed a significant excess of heterozygotes compared to Hardy-Weinberg expectations at that point (mean *I  = *−0.20±0.052; Figure 2e).

Allele frequencies changed significantly between generations in only three cases out of 72 ( = 18 replicates ×4 generations). The mean (± SE) overall change in allele frequencies between the start and generation eight was −0.010±0.027).

The assortative mating that occurred was not based on colour, as females of both colour morphs mated as readily with black as with brown males (9 brown vs. 12 black, X^2^ = 0.43, P = 0.51, df = 1). The time to mating was not significantly different between the different morphs (brown: mean ± SE = 308±111 sec, black: 387±96 sec; U = 39, P = 0.28, Mann-Whitney U-test); however, the duration of mating was to a certain extent longer for both types of females mated to black males compared to brown males (brown: 454±51 sec; black: 617±44 sec; U = 24.3, P = 0.034, Mann-Whitney U-test). This is in part due to one mating with a black male that was about 65% longer than the mean duration. If this mating is excluded the difference is no longer significant (brown: 454±51 sec; black: 585±39 sec, U = 24.5, P = 0.058, Mann-Whitney U-test).

Colour seemed to be neutral both with regard to host preference and host performance, as the mean number of eggs laid on the two substrates did not differ between females of the two colour morphs (brown: 65.9±5.33; black: 61.0±4.61, U = 48.5, P = 0.70, Mann-Whitney U-test), nor did the mean number of individuals hatched from the two substrates differ between the two morphs (brown: 61.3±5.03; black: 56.7±4.35, U = 42.5, P = 0.42, Mann-Whitney U-test). No difference in larval performance could be found as egg to adult survival was the same for both female types on both substrates (*F*_1, 18_ = 0.00052, *P* = 0.98 (on M), *F*_1, 18_ = 0.013, *P* = 0.91 (on BE), ANCOVA with the number of eggs as covariate).

The deficit of heterozygotes in the early generations did not correspond to a lower viability of heterozygotes, as evident when comparing the number of adults that emerged of the different morphs (brown morph 548.0±45.1,; intermediate 512.0±28.0; black 459.3±4.09, mean ± SE number of adults, *P* = 0.67, Kruskal-Wallis test). No difference in larval performance could be found as egg to adult survival was the same for all three morphs on both substrates (*F*_2, 2_ = 0.27, *P* = 0.79 (on BE), *F*_2, 2_ = 4.58, *P* = 0.18 (on M), ANCOVA with the number of eggs as covariate).

## Discussion

The bruchid beetle *Callosobruchus maculatus* is known to be fairly plastic when it comes to host use. However, seed beetles from the same group are often specialized and predominantly use closely related plant taxa from a single host tribe [21]. We found that lines of *C. maculatus* raised on a novel food resource (mung beans) expanded their host range and evolved a new preference for this resource over only ten generations of selection, and in the same short time span also showed reduced preference for the ancestral host (black-eyed beans). Since we did not obtain this pattern after only one generation on a novel resource, the result is unlikely to be a result of larval conditioning or maternal effects. Maternal effects are known to exist in insects, and mothers may influence their progeny's life history traits in many ways; the environment that the mother experiences can influence oviposition decisions and host suitability for the offspring [22]. In this study, maternal effects could potentially influence preference and performance on the two hosts, but our results suggest that this is not the case. We also found significant deviations from Hardy-Weinberg expectations in terms of a strong deficit of heterozygotes in the first two generations, while this changed to a significant excess of heterozygotes later on. The most likely explanation for the result is assortative mating based on resource use, as we were unable to find any selection acting on heterozygotes.

Colour morph was found to be neutral in the base population relative to the traits tested, i.e., host preference, host performance and mate choice, suggesting that in natural, unselected populations, colour is not closely linked to the above mentioned traits. In our study, we selected the lines in a way to create a close linkage between colour and host preference, and thus mate choice. However since matings were performed in dark cabinets we find it unlikely that colour would confound the results in this study. It would be more likely that mate choice is based on some other, nonvisual cue, such as smell for example.

In short, we found that preference for a food resource can coevolve with a mating preference as models suggest, but that this is not stable, as we were unable to detect selection acting against hybridization. Assortative mating alone cannot maintain reproductive barriers in this case, a result consistent with theoretical expectations of assortative mating and disruptive selection having to work in concert to hinder gene flow between diverging populations [1].

Our results are in accordance with Wasserman and Futuyma [23] who found a positive response to selection for ovipositional preferences after eleven generations of selection in the same species and Messina et al. [24] who also demonstrated a change in oviposition preferences in seed beetle lines switched to new hosts. Since host performance did not evolve alongside host preference, no fitness consequences seem to have resulted from the selection. This in turn supports the findings of Fox [25] who found no positive genetic correlation between host preference and host performance in the same bruchid beetle. The lack of dependence between the two traits was also found by Forister [26] and Magalhaes et al. [27]. Thus, our novel result is that a difference in fitness on the two alternative host plants is not necessary to induce assortative mating, provided that mating at the time of selection takes place on the food resource.

There are three possible explanations for the deficiency of heterozygotes in the first generations selection, assortative mating and inbreeding. Several lines of evidence suggest that selection is not important in this case. First, we found no fitness differences between the heterozygotes and the homozygotes. Second, the change in allele frequencies was approximately what can be expected by drift. Changes in the frequency of heterozygotes over the different periods are a result of a redistribution of alleles among homozygotes and heterozygotes as a result of changing mating patterns, and not due to a change in allele frequencies as a result of selection. Third, inbreeding could also cause deviations from Hardy-Weinberg expectations, for example, if the new populations were founded by only a few individuals, i.e. if the effective population size was very low in the tests. However, if this were the case, then both a deficit and an excess in heterozygotes would be expected. This leaves us with assortative mating as the most likely explanation.

The relationship between mating- and food preference was not immediately broken down as a result of gene flow between the different morphs, but instead lasted for a couple of generations. This suggests that partial reproductive isolation can arise as a consequence of adaptation/habituation to different types of food resources. However, this effect disappeared in most lines by generation three and after that, we found clear signs of disassortative mating, possibly reflecting inbreeding avoidance. Assortative mating as a result of differential selection has been shown before in different contexts [28]. These studies were all selection experiments for different environmental conditions such as selection for positive or negative geotaxis in houseflies, selection for differential temperature and humidity in *Drosophila*, and selection for spatiotemporal habitats, phototactic and geotactic behaviour in *Drosophila* [29]-[31]. In all of these cases, the strains selected on different hosts showed signs of reproductive isolation. The experiments performed, both previous and ours, also support the assumption that mating preference readily evolves as a by-product of host/habitat preference.

Udovic [7] showed that sympatric speciation only occurs if the cumulative strength of selection against hybrids (s) and the strength of assortative mating (α) is larger than the threshold strength for each of them to cause complete reproductive isolation when acting in isolation, i.e. s + α >1. In our experiment, selection against hybrids is lacking and as assortative mating cannot take a value larger than one, this threshold is not passed. As a consequence, complete reproductive isolation could not be reached. This may represent a valid explanation for rapid breakdown of the partial reproductive isolation obtained in the two first generations and the return to random mating between the two distinct morphs.

An important finding is that there was considerable variation among replicates in the extent of assortative mating. For example, replicate 8 had a significant excess of heterozygotes at generations one to four, but not in generation eight. On the other hand, replicate 3 did not have an excess at all, but instead developed a significant deficit over time (see Figure 2a–e). This suggests that the evolution of assortative mating in this system has a strong stochastic component, probably as a result of which particular genes were present at the time the experiment started. In a more general sense, this implies that only a fraction of the host shifts will eventually result in a genetic differentiation between populations. A number of conditions need to be met for speciation via host-shift to occur, such as evolution of assortative mating and selection against hybrids, but if these are fulfilled, speciation can be rapid. In our case, assortative mating evolved rapidly, but since there was no selection against hybrids, the linkage between food preference and mating preference was quickly destroyed by recombination. If our experiment represents a common situation in nature, we can expect to see a number of “speciation experiments”, with many failures due to the fact that all necessary conditions are not fulfilled.

The variation among lines could be a result of the low effective population sizes used in the selection part of the experiment, since a low effective population size would result in stronger stochastic effects than in a larger population. Low effective population size could also lead to inbreeding and subsequent loss of fecundity and hatching success. However, fecundity and hatching success were at the level found in the control lines, and hence no obvious inbreeding effects were observed.

In conclusion, we have shown that the predicted association between food and mate preference in phytophagous insects can readily evolve given selection for food preference in allopatry, but the maintenance of reproductive isolation after secondary contact is ultimately dependent on selection acting against hybrids, and/or mating taking place at the food resource. Thus, the establishment and maintenance of linkage disequilibrium between pre- and post zygotic isolation is required for speciation to proceed to completion. This is in accordance with most models of speciation assuming the given ecological circumstances.
